# The emotional and social burden of heart failure: integrating physicians’, patients’, and caregivers’ perspectives through narrative medicine

**DOI:** 10.1186/s12872-020-01809-2

**Published:** 2020-12-12

**Authors:** Marco Testa, Antonietta Cappuccio, Maura Latella, Silvia Napolitano, Massimo Milli, Massimo Volpe, Maria Giulia Marini, TRUST participants

**Affiliations:** 1 Cardiology Unit, Sant’Andrea Hospital, Rome, Italy; 2 Area Sanità e Salute di Fondazione ISTUD, via Paolo Lomazzo 19, 20124 Milan, Italy; 3 Medical Department, Novartis Farma, Origgio, Italy; 4 Cardiology Unit, Santa Maria Nuova Firenze Hospital, Florence, Italy; 5 School of Medicine and Psychology, University of Rome “Sapienza”, Rome, Italy; 6 IRCCS Neuromed, Pozzilli, Italy

**Keywords:** Heart failure, Narrative medicine, Doctor-patient relationship, Quality of life, Informal caregiver

## Abstract

**Background:**

The The Roadmap Using Story Telling project used a narrative medicine (NM) framework to assess the perspectives of people with heart failure (HF), their informal caregivers and HF specialists of the impact of HF on the daily life of patients and their carers.

**Methods:**

Italian HF specialists participated on a voluntary basis, completing their own narratives, and inviting patients and their caregivers to write anonymously about their experiences, all on a dedicated online platform. The narratives were analyzed according to standard NM methodology.

**Results:**

82 narratives were collected from patients, 61 from caregivers, and 104 from HF specialists. Analysis of the three points of view revealed the extent of the burden of illness on the entire family, particularly that of the caregiver. The impact was mainly experienced as emotional and social limitations in patients’ and their caregivers’ daily lives. The analysis of all three points of view highlighted a strong difference between how HF is perceived by patients, caregivers, and HF specialists.

**Conclusions:**

This NM project illustrates the complex issues of living with HF and gave insights to integrate three different perspectives into the HF pathway of care.

## Key points for decision makers

- Patients and their informal caregivers have a strong need to be heard; narrative medicine provides this opportunity.
- Two parallel lives were disclosed: patients’ lives, which are affected by their HF-related physical limitations, and lives of informal caregivers, burdened emotionally by caring for a family member.
- NM offers HF specialists the opportunity to better understand the patient experiences of HF, and an opportunity to actively recognize the role of the caregiver and educate both.

## Background

Heart Failure (HF) is a progressive chronic disease that needs long-term management. It affects 1–2% of the European population and about 10% of people between 75 and 80 years old [1]. HF incidence is currently increasing, mainly due to innovative therapies and improved survival from myocardial infarction [2].

HF standard treatment requires the patient to take several drugs on a daily basis; then, it is not only difficult to adhere to but further burdens patients’ and caregivers’ daily lives, negatively affecting their quality of life (QoL) [3, 4]. Moreover, most people with HF have comorbidities, including hypertension (58.4%), atrial fibrillation (25.3%), chronic kidney disease (51.4%), and dyslipidemia (44.6%) [5], enhancing patients’ and caregivers’ burden.

HF symptoms in patients have been found to be associated with strain in their caregivers [6]. Indeed, the risk of depression and anxiety, as well as financial loss, increases over time in families as a direct consequence of providing care for a person with deterioriating health; this is especially true for female informal caregivers [7]. Risk of hospital readmission rates for people with HF are correlated with lack of social support [8], suggesting the important role of the family environment in their care. The need for research into clinical- and person-oriented outcomes of both the person with HF and their caregiver(s) has been recognized [6].

Narrative medicine (NM) is based on the analysis of narratives of illness experiences [9]. NM pursues the integration of the disease-centered approach, focusing on clinical aspects, with the illness- and sickness-centered approaches, respectively concerning personal coping and social perception of a condition [10]. NM is considered informative since integrating all the perspectives involved in the care pathway helps to reveal common issues as well as possible interventions or solutions about experiencing a condition [9, 11–13]. The parallel chart and the illness plot represent the main NM tools, respectively dedicated to healthcare professionals and to patients and caregivers [14, 15]. Recent studies demonstrated the advantages of applying the parallel chart in exploring healthcare professionals’ point of view on the care pathway for chronic conditions (i.e. chronic obstructive pulmonary disease, COPD) and doctor-patient relationships [16, 17]. Similarly, studies in other chronic conditions indicated illness plots dedicated to patients and caregivers to be a source of information on personal coping with the disease, and on how patients and their families rearrange their lives after diagnosis [18–20].

Through NM, the TRUST (The Roadmap Using Story Telling) project mainly aimed to investigate the perspectives of people with HF, their informal caregivers and HF specialists on the impact of this condition on their daily experience.

## Methods

### Research design and setting

The cross-sectional TRUST project was conducted at 21 HF clinics across Italy. Beginning May 2018, 25 HF specialists working at these clinics were invited to take part in a voluntary training session on NM on the methods and aims of the project. Then, one specialist from each of the 21 HF clinics which decided to participate to the project invited patients with HF and their informal caregivers to participate, providing them with information materials about the TRUST project.

A board composed of two Italian HF specialists and one patient reviewed the NM tools and patient informed consent forms, developed by the “Istituto Studi Direzionali” (Institute of Management Studies, ISTUD) Foundation [21], and then adapted them to the project’s purposes. The NM tools used were followed by a sociodemographic survey (Additional file 1: Appendix 1) and consisted in a semi-structured parallel chart for physicians and two different illness plots for patients and caregivers; the prompts were composed of brief sentences characterized by evocative and open words with the aim to ease individual expression (Additional file 2: Appendix 2) and specifically designed to overcome writer’s block [22]. All narratives were written in Italian.

### Data collection

From June to November 2018, physicians completed their parallel charts: the only inclusion criterion was writing about a person with a confirmed HF diagnosis whom they had seen at least twice, with no restrictions in terms of disease severity or other clinical parameters. Patients and caregivers independently and anonymously provided their narratives on a dedicated online platform, accessible through the project’s webpage www.medicinanarrativa.eu/trust. The platform is designed to facilitate research in the healthcare sector, and it is fully compliant with the General Data Protection Regulation (GDPR) of the European Union 2016/679. Only researchers had access to the survey responses and deleted any identifiable element in narratives; all authors were blinded to the participants’ identities.

### Ethical considerations

Physicians, patients and caregivers shared their narratives anonymously; consequently, patients described in parallel charts could be different from those participating in the project. Furthermore, physicians or researchers were not able to identify any potential relationship between them. Prior to writing their narratives, participants provided online written informed consent after being informed on the project’s aims and confidential data handling procedures, according to the Italian Law 196/2003 on Privacy and Safeguarding of Sensitive Data and the GDPR of the European Union 2016/679. The project was approved by the Institutional Review Board of the Santa Maria Nuova Hospital (Florence, Italy).

### Data and narrative analysis

Researchers analyzed the sociodemographic data through descriptive statistics. Raw and anonymous narratives were downloaded as a Microsoft Excel spreadsheet and independently analyzed by three researchers from ISTUD Foundation, different for academic background, with the support of the qualitative data analysis software NVivo10 (QSR International), which allows a thorough analysis of recurring words and semantic expressions. Researchers employed open interpretive coding to identify emerging topics.

Before sharing their narrative, patients were asked to describe HF with a metaphor or an image: employed metaphors were retrospectively divided into groups to facilitate interpretation. Furthemore, narratives were analyzed through three classification employed in NM, identified by authors as the most suitable to highglight the several aspects of HF experience and caregiving: (a) Kleinman’s classification [10], distinguishing between disease-centered narratives, focusing on the clinical evolution of the condition and employing a technical language, and illness- and sickness-centered narratives, respectively concerning the personal experience and the social meaning of the condition; (b) Plutchik’s wheel of emotions [23], based on eight primary emotions (joy, trust, surprise, anticipation, sadness, fear, anger, and disgust) and their different degrees, named secondary emotions; (c) Frank’s classification [24], discerning between chaos narratives, characterized by a fragmented sequentiality and moods of confusion and pain, restitution narratives, reporting healing expectations and the return to a previous health situation, and quest narratives, expressiong the search for a meaning of the illness experience.

## Results

### Sociodemographic characteristics

Eighty-two narratives were collected from patients, 61 from caregivers, and 104 from HF specialists, for a total of 247 narratives (Additional file 3: Appendix 3). Seventy-five percent of people with HF were male (75%) with a mean age of 68 years; most caregivers were patients’ wives (47%) or daughters (35%), respectively with a mean age of 60 and 46 years. Seventy-one percent of patients were pensioners and 37% of caregivers were employed. The sociodemographic characteristics of patients and caregivers were similar to those of the general Italian population, except for education attainment level, which was higher than the Italian average [25]. HF specialists’ parallel charts described patients with a similar sociodemographic profile to that of participating patients (Additional file 3: Appendix 3).

### Management of the condition

HF diagnosis in patients frequently occurred before 60 years of age; the mean disease duration was 10 years for patients and as described by caregivers, and 8 years according to physicians (Table 1). HF specialists reported other cardiovascular comorbidities affecting 63% of their patients. On average, families had to cover 37 km to reach the cardiology center, with follow-up every 6 months or more frequently (86%). At home, disease management included the administration, on average, of 8 different drugs per day across seven different times. Twenty-seven percent of patients showed class II HF according to New York Heart Association (NYHA) classification, while 26% reported a NYHA class III; however, physicians described the people with HF as suffering from more severe HF (44% class II, 34% class III). Although patients and caregivers know the patient's ejection fraction (EF) with exam withdrawal, respectively 16% and 17%, when asked, reported to not know the answer (Table 1).

**Table 1:** Disease management and clinical characteristics of patients with HF reported by patients, HF specialists, and caregivers

	Patients (*N* = 82)	Patients described by physicians (*N* = 104)	Patients described by caregivers (*N* = 61)
Age (mean ± SD), years	57 ± 3	57 ± 3	–
Disease duration (mean ± SD), years	10 ± 2	8 ± 1	10 ± 3
Recurrence of specialist visits, n (%)	(*n* = 73)	–	(*n* = 52)
≥ 1 in 6 months	28 (38)	–	25 (48)
1 per 6 months	35 (48)	–	23 (44)
1 per year	8 (11)	–	2 (4)
< 1 per year	3 (4)	–	1 (2)
Just in emergency cases	–	–	1 (2)
NYHA class, n (%)	(*n* = 73)	(*n* = 92)	(*n* = 53)
I	18 (25)	4 (4)	17 (32)
II	27 (37)	44 (48)	16 (30)
III	26 (36)	34 (37)	18 (34)
IV	2 (2)	10 (11)	2 (4)
Ejection fraction, n (%)	(*n* = 73)	(*n* = 102)	(*n* = 52)
> 40% (HF-pEF)	18 (25)	17 (17)	14 (27)
< 40% (HF-rEF)	39 (53)	85 (83)	21 (40)
I don’t know	16 (22)	–	17 (33)

*HF* heart failure, *NYHA* New York Heart Association, *pEF* preserved ejection fraction, *rEF* reduced EF, *SD* standard deviation

### Analysis of narratives

#### Word frequency

The analysis of the 100 most frequently used words in narratives showed differences across the three considered perspectives. Patients frequently used words evoking previous life conditions and expressing fatigue, tiredness, and difficulty carrying out activities that before were considered normal, such as walking, working, and climbing stairs (e.g. «Before my illness I was always active, I’d walk at least 2 h a day and I had a balanced diet. The only unhealthy thing I did was smoking»).

The word “fear” was used more commonly by caregivers than by patients, suggesting concern for QoL and life expectancy of the patients they cared for. Words related to the medical domain (e.g. “physicians”, “therapy”, “follow-up visit”) were also present.

Parallel charts highlighted the improvements obtained with treatments; thus, caregivers were represented as background figures or helpers in the event that patients should fail to comply with treatments. In most of their narratives, physicians showed trust at diagnosis but felt an urge to reassure their patients (e.g. «None of them should blame him/herselves, but they all had to undertake to follow the doctor’s instructions from diagnosis onwards»). Physicians proved to be aware of the importance of knowing how to actively and thoroughly listen to their patients, not only in the clinical domain but also in the speheres of emotions and everyday life planning.

#### HF social burden on patients and caregivers

All narratives were detailed on patients’ QoL (Table 2). HF consequences were so burdensome that only 26% of patients and 16% of caregivers stated that they had returned to their usual life (e.g. «I’ve had to reduce my working hours and ask my children and family for help. Today we spend much of our time at home. We don’t go anywhere»). Among the three groups of participants to the project, female caregivers mainly denounced their caregiving burden: in 55% of narratives, the duration of caregiving was reported to exceed 8 h per day. Furthermore, 34% of caregivers considered impossible resuming activities previous to HF diagnosis as it would imply leaving patients alone.

**Table 2:** Patients’, physicians’ and caregivers’ perception of the impact of HF on daily activities

	Patients	Informal caregivers	Patients, as described by physicians	Patients, as described by caregivers
Impact on work, n (%)	(*n* = 33)	(*n* = 21)		
No changes	6 (18)	5 (24)	–	–
Feeling disadvantaged at work	3 (9)	1 (5)	–	–
Limiting activities at work	14 (42)	9 (43)	–	–
Work interrupted	10 (30)	6 (29)	–	–
Spare-time activities before the diagnosis of HF, n (%)	(*n* = 70)	(*n* = 33)	(*n* = 79)	
Social Life (i.e. dinner with friends, theatre, etc.)	32 (46)	19 (58)	39 (49)	–
Sport	17 (24)	5 (15)	10 (13)	–
Work and little spare time	10 (14)	–	13 (16)	–
Taking care of the family	7 (10)	8 (24)	13 (16)	–
Gardening	4 (6)	1 (3)	4 (5)	–
Impact on daily activities today, n (%)	(*n* = 69)	(*n* = 32)	(*n* = 74)	(*n* = 44)
Social life (i.e. friends, theatre, etc.)	18 (26)	5 (16)	34 (46)	5 (11)
Light physical activities (i.e. bike, walk, etc.)	14 (20)	–	14 (19)	
Reading and watching TV	7 (10)	–	1 (1)	
Taking care of the family	5 (7)	7 (22)	4 (5)	
Art (i.e. painting, music, etc.)	3 (5)	–	5 (7)	
Limited activities	15 (22)	–	10 (14)	18 (41)
Impossible to restore activities	6 (9)	9 (28)	6 (8)	16 (36)
Activities not restored due to fear	1 (1)	11 (34)	–	5 (11)

*HF* heart failure

Considering Kleinman’s classification [10], narratives were mostly illness-centered (96% for patients, 100% for caregivers, 96% for physicians).

#### HF emotional impact

**Fig. 1:**
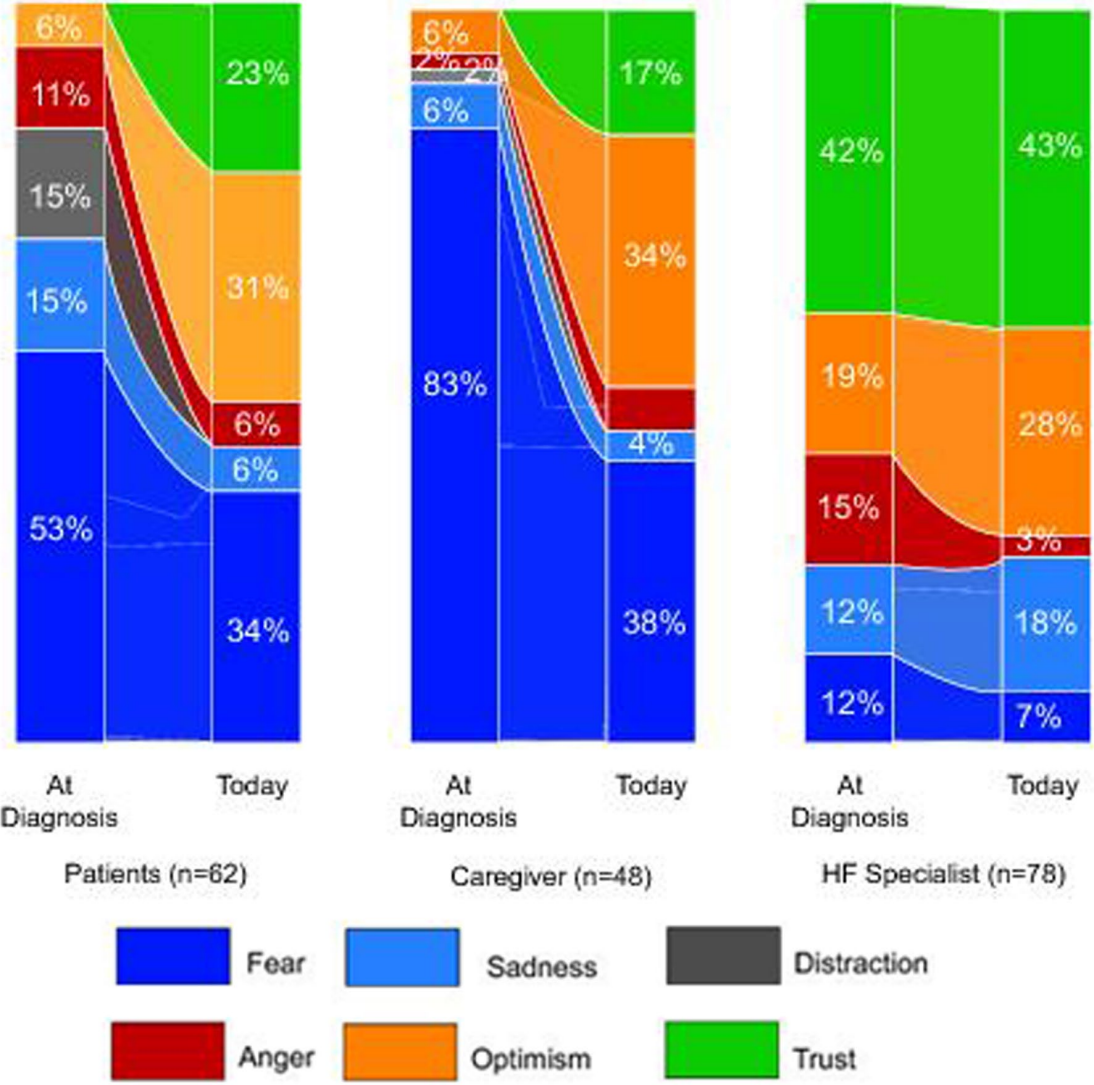
The emotional impact of HF reported by patients, informal caregivers, and HF specialists: a comparison of the emotions felt at diagnosis (via recall) versus those felt at the time of providing their narrative. Data are reported as proportion of patients/caregivers/HF specialists. *HF* heart failure

HF emotional impact on participants was also investigated through the Plutchik’s wheel of emotions [23]. At diagnosis, physicians’ prevalent emotions were trust and optimism (61%), and this positivity was still present (71%) during the writing of parallel charts (Fig. 1). On the other hand, patients reported fear (53%) and sadness (15%) as the most frequent emotions at diagnosis; this emotional impact was also confirmed by both caregivers and physicians.

Although in narratives patients reported current emotions such as optimism (31%) and trust (23%), 34% of them still felt fear, suggesting that they continue to feel afraid about the condition. Similarly, more than 80% of caregivers described having felt fear and anguish at diagnosis, and these emotions remained in a significant proportion (38%) at the time of writing.

Twenty-one percent of patients and 15% of caregivers described the future as frightening, and 17% and 23%, respectively, declared to avoid thinking about it; indeed, in narratives 41% of caregivers referred to the fear of the sudden death of their loved ones.

#### Perception of the condition

**Fig. 2:**
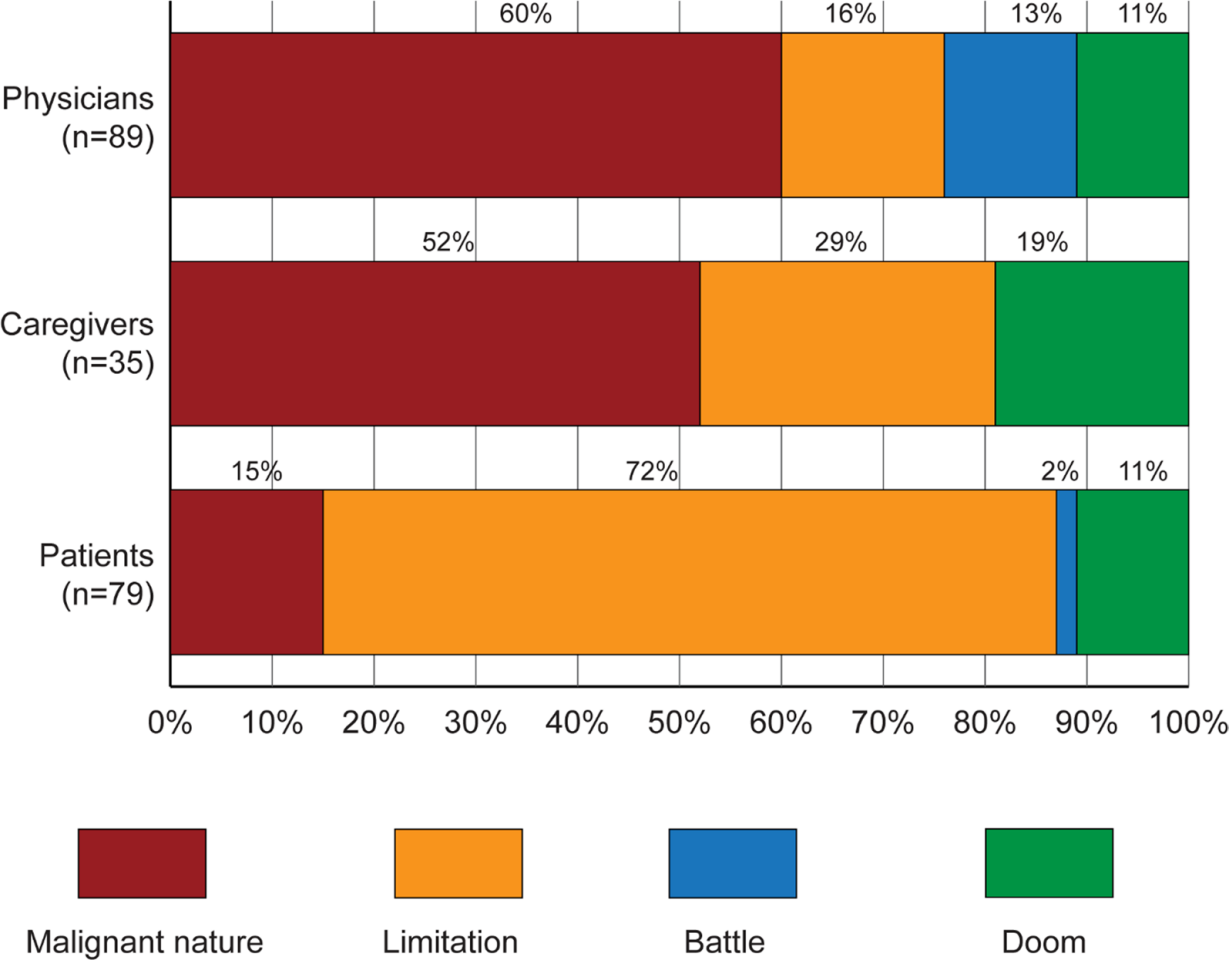
Language analysis of participants’ metaphors to describe HF. Data are reported as proportion of patients/caregivers/HF specialists. *HF* heart failure

The metaphors used by participants to define HF (Fig. 2) were grouped into four main classes to facilitate interpretation: (a) *malignant nature* metaphors, relating to something frightful or unpredictable (e.g. «volcano eruption»); (b) *limitation* metaphors, in which the condition is perceived as disabling (e.g. «a very fast car without fuel»); (c) *fight* metaphors, where HF is seen as an enemy (e.g. «a trench war»); (d) *threat* metaphors, in which danger is the main feature (e.g. «the sword of Damocles»). Seventy-two percent of patients expressed *limitation* metaphors, 52% of caregivers *evil nature* metaphors (e.g., «slowness» of life), as did the 60% of physicians (e.g. «earthquake», «panther»); 13% of physicians also used *fight* metaphors (e.g., «trench war»).

#### Disease awareness

**Fig. 3:**
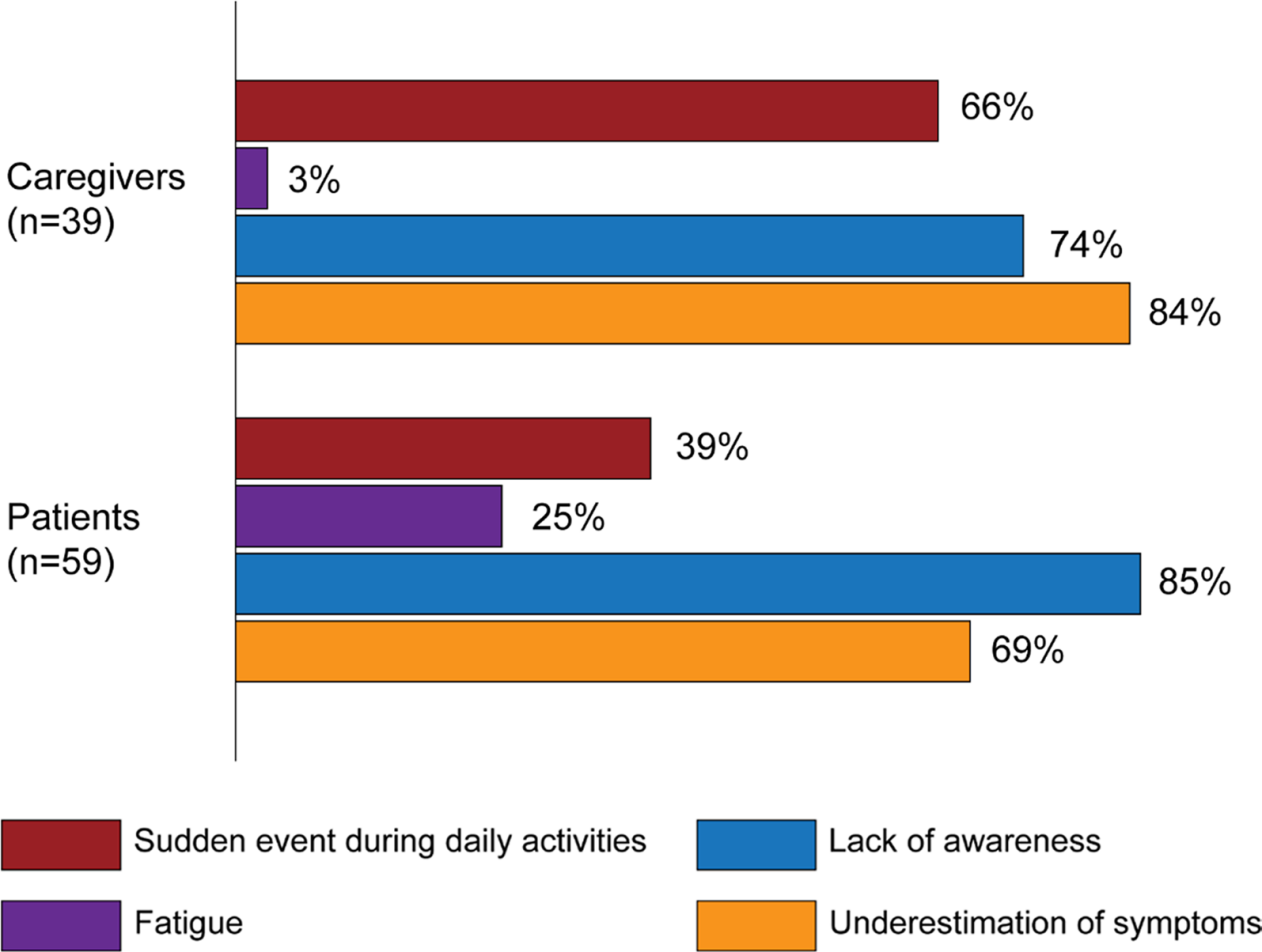
Awareness of the disease symptoms as described in patients’ and caregivers’ narratives

Sixty-nine percent of patients and 84% of caregivers stated that they had not initially recognized the first symptoms of the disease; 44% of physicians also reported this underestimation. Participants described no initial symptoms since—according to 39% of patients and 66% of caregivers—HF suddenly occurred during daily activities, although 25% of patients reported having noticed unusual fatigue before the diagnosis (Fig. 3). In fact, 55% of physicians reported first meeting the person with HF in an emergency situation. Furthermore, both patients’ and caregivers’ narratives showed a lack of awareness about the condition (85% of patients and 74% of caregivers) exemplified by their misuse of clinical terms and poor knowledge about what exactly HF is (Fig. 3).

#### Doctor–patient relationship

Relationships in the care pathway were clustered in three main ways: “easy” relations, when described as comfortable and trustworthy, “difficult”, when described as unsatisfactory, and “evolved”, when initially difficult but with a positive evolution.

**Fig. 4:**
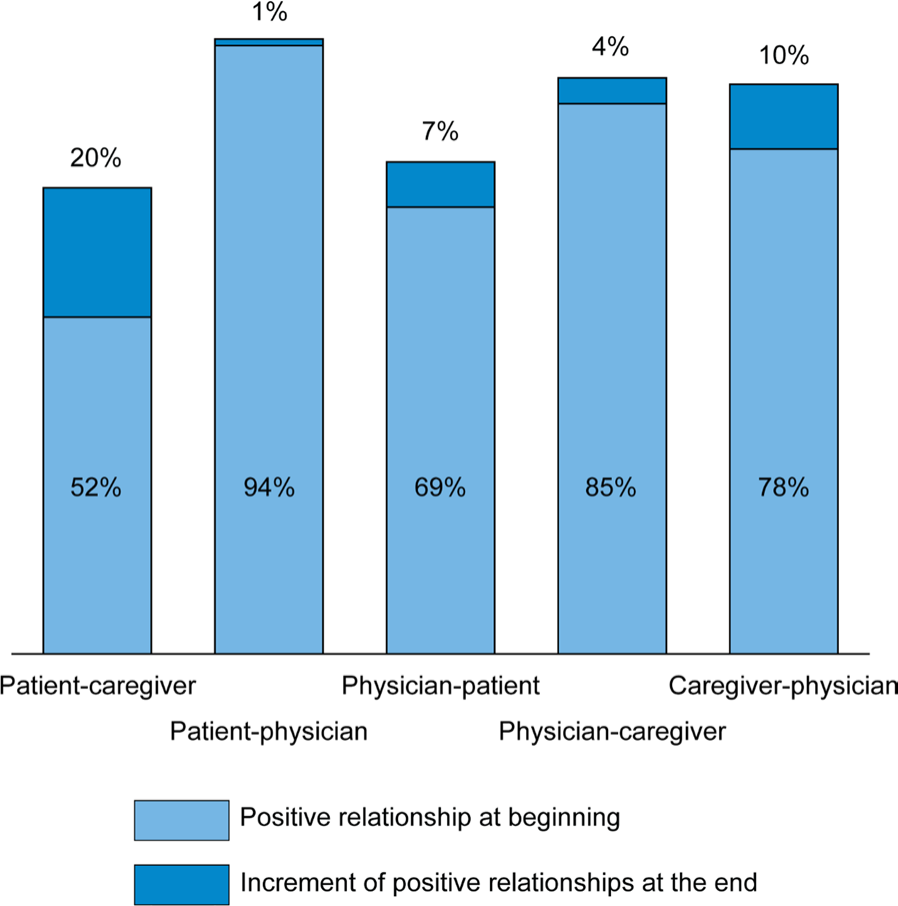
Positive relationships involving patients, caregivers, and HF specialists from the patients’, caregivers’, and physicians’ perspectives, at the beginning of the narrative (light blue) and the incremental increase due to evolved relationships at the end of the narrative (blue)

Physicians established good relationships with patients and their families (Fig. 4); furthermore, from their perspective, 7% of doctor-patient relationships that were initially difficult improved over time. The highest proportion of difficult relationships early after diagnosis reported by patients were for those between patient and caregiver when the caregiver was a family member (48%). Patients often described these caregivers as being more afraid than necessary, and as annoying to the patient, who often desired more autonomy.

#### Perception of treatment

Therapies were described as effective and were often considered to have contributed to positive relationships between patients and caregivers. Generally, both patients and caregivers were satisfied with patient treatment (more than 80% of each considered them effective or very effective), while surgery (e.g. implantable devices, heart transplant), cited by 20% and 6% respectively, was considered the most critical treatment in terms of both risk and effect on outcomes. On the other hand, HF specialists perceived the treatment plan for patients as complex and burdensome in 21% of the cases, more often than patients and their caregivers.

#### Participation in NM project

Patients, physicians and caregivers reported their general appreciation for writing about their experience, and sharing their own narratives was perceived as a liberation and an opportunity to reflect (e.g. «I was pleased I was able to describe our experience in the hope that it may be useful; indeed, I wanted to thank you for giving me this opportunity»). Twelve percent of narratives stated that sharing the experience was difficult (e.g. «Remembering the single moments arouses a feeling of emotional suffering for a situation that is still unsolved and that presents an objective uncertainty for the future of all our family»).

## Discussion

The TRUST project aimed to explore the living with HF through the integration of patients’, caregivers’, and HF specialists’ perspectives. Firstly, the large number of narratives collected for this project can be considered as an excellent result, highlighting the need of patients and their caregivers alike to be listened to. Indeed, participating in the project was rated as a positive experience by about 90% of patients and caregivers, and an even higher percentage of physicians perceived the task of writing as a way to reflect on their work.

Results show that HF strongly limits the life of both patients and their caregivers. Narratives revealed two parallel lives: the life of patients, which is physically limited, and that of informal caregivers, which is affected by the need to look after a family member. A substantial proportion of patients adopted hobbies, like playing cards or reading, in place of strenuous activities such as sport. A strong emotional impact of HF emerged in terms of anxiety and fear of sudden death for both patients and caregivers. This anguish led to additional limitations to activities, and caregivers reported being not only responsible for co-ordinating the patient’s complex therapy plan and medical visits, but also having to be constantly in close proximity to the patient due to their deep fear of sudden worsening of the condition. Furthermore, most caregivers were women, partners, and daughters: their narratives exemplified the sacrifices they made. HF emotional impact on patients and their families agrees with a previous study, in which caregiving demands have been related to depression in the caregiver [26]. Both patients and caregivers had to change their daily lives to cope with this new condition.

Almost all participants wrote about HF as an “illness”, according to our analysis using Kleinman’s classification [10]. These results contrast with those seen for other chronic diseases, for example, COPD [19], in which patients report sickness-centered narratives. Therefore, although some people with HF had engaged in harmful behaviors (i.e. smoking, consuming alcohol, over-eating) that may have contributed to HF development, they did not feel judged for the onset of HF. Furthermore, beyond cardiologists, these results could be shared with General Practitioners (GPs), which could empower the illness-centered relationship to establish an effective communication with people with HF, so to increase patients’ disease awareness and improve access to care.

Relationships were positively described and patients often expressed gratefulness to their doctors and for effective therapies, as confirmed by the high percentage of restitution narratives, according to Frank’s classification [24]. Our results are in contrast with a recent Swedish study, which showed that caregivers felt unrecognized for their role in HF management [27], suggesting that caregivers may have difficulty in establishing a positive relationship with the physician. HF care is particularly challenging, not only for the elderly age of occurrence and frequent comorbidities, but also for the high number of different drugs per day patients have to consume. However, it is interesting to notice that the complexity of the treatment plan was considered more burdensome by the physicians than by either the patient or their caregiver. A recent study in patients with COPD demonstrated a link between the physicians’ style of writing about their relationship with patients and the quality of care of their patients [28]. In that study, participating physicians wrote illness-centered narratives. We have to notice that our results could be biased towards a positive physician–patient relationship because the HF specialists participating in the TRUST project were already attentive to their relationship with patients.

Both caregivers and HF patients frequently avoided seeking the help of a HF specialist or other healthcare professionals until the dramatic worsening of the condition, even if they had recognized unusual fatigue. Patients’ narratives included many elements of confusion; a lack of knowledge of HF emerged from their experiences, reflected by their deep feelings of fear and anguish, and those of their caregivers.

Moreover, even with a recent diagnosis, almost 30% of patients and their families did not know their EF, suggesting that they probably ignored the severity of their condition. Indeed, families’ poor health literacy and knowledge of HF have been recognized in previous studies [29, 30], especially in terms of understanding specific terminology; moreover, recent evidence [31] showed that low levels of literacy and limited disease awareness are influenced by age-related factors and communication information. Nevertheless, participating patients and caregivers had educational attainment levels higher than the Italian standard, so they had the necessary means to understand HF course. Interestingly, scarce knowledge of HF was shown even by participants involved in the ‘HF Awareness Day’ initiative launched by the European Society of Cardiology HF Association [30], suggesting that their desire to be involved in such initiatives was not correlated with higher acceptance or awareness of the disease. What could be inferred from their participation in the HF Awareness Day initiative was their greater desire for effective care, and ultimately complete healing. The analysis of metaphors revealed a large difference between patients and physicians in how they defined the condition (essentially an “internal” understanding or knowledge of disease). While doctors expressed awareness of the inevitable progression of HF, using “malignant nature” metaphors, patients were mainly focused on the “limitations” they experienced because of HF (Fig. 2). Furthermore, the high level of fear and anguish felt by patients and the caring attitude of physicians could have contributed to the doctors’ lack of communication of the severe disease prognosis. Further studies could adequately address this point; however, an Italian study [32] demonstrated the effectiveness of including NM training in the education pathway of cardiology specialists.

Against this backdrop, the informal caregiver deserves a multidisciplinary attention: the stress of caregiving affects not only the caregiver’s wellbeing but that of the whole family. To improve the caregiver’s condition, it would be useful to strengthen the therapeutic alliance of the physician–patient-caregiver triad, by acting in the following directions:

- Providing individual psychological support such as brief or extensive counseling, organized in such a way as not to require an excessive time investment or constitute an additional burden on the subject’s psychophysical resources;
- Promoting caregiver interaction within support groups, in order to stimulate and facilitate elaborative/transformative processes allowing the acquisition of new strategies in the daily management of the patient and preventing social isolation;
- Providing adequate information to the patient and caregiver at the time of communicating the diagnosis, so as to allow adequate understanding of the disease and make the necessary changes to their behavior.

Pinpointing a limitation of this project, that could be traced in the fact that all data were gathered by self-report. The large number of narratives collected and the integration of different points of view may help reduce to some extent the possible bias of using a qualitative methodology. Two other possible biases can be highlighted: the first, the high level of educational attainment among participating patients and caregivers. The second, we did not get information on caregivers’ health conditions, which may represent an overlapping factor in HF burden; moreover, caregivers may have further issue of inflating HF burden if they were financially supported by their relatives with HF before the diagnosis.

## Conclusions

Our NM project enabled us to describe the profile of those living with HF and those taking care of people with HF in Italy. Integrating patients’, caregivers’, and HF specialists’ perspectives, the burden of illness on the entire family emerged from understanding the key role of the caregiver in the daily management of the complex care of HF. The impact described in the narratives was mainly focused on the emotional and social limitations of both patients’ and caregivers’ daily lives, impeding their work activities and impacting on their hobbies and relational sphere.

The strong presence of fear and anguish in patients’ and caregivers’ narratives were probably a consequence of their general lack of knowledge and understanding of HF. Indeed, the collected narratives unveiled different levels of HF awareness: this lack of literacy is mainly caused by scarce communication, failure to capture the meaning of illness experience and to see caregivers as fundamental elements in the care pathway, able to contribute to improve the awareness of the condition and coping strategies.

The application of NM could be considered an effective tool for integrating the different perspectives on living with HF, and to strengthen the triad of care and the therapeutic alliance.

## Supplementary Information

**Additional file 1: Appendix 1** Sociodemographic survey for participants. The Appendix provides the track of thesociodemographic surveys employed to collect data of, respectively, patients, caregivers, andhealthcare professionals involved in the research.

**Additional file 2: Appendix 2** Illness plots and parallel charts. Narrative prompts used in the project and specifically designed by the board and the researchers of the ISTUD Foundation. The brackets stand for the space where the participants could write about their experiences..

**Additional file 3: Appendix 3** Sociodemographic data of participants. Sociodemographic data of patients, caregiver, HF specialists who participated to the project and of patients described by physicians in their narratives.

## Data Availability

All datasets used and analysed during the current research are available in Italian from the corresponding author upon reasonable request.

## Abbreviations

EF: Ejection fraction
GPs: General practitioners
HF: Heart failure
ISTUD: Istituto Studi Direzionali
NM: Narrative medicine
NYHA: New York Heart Association
QoL: Quality of life
TRUST: The roadmap using story telling
