# Supplementary material for: The emotional and social burden of heart failure: integrating physicians’, patients’, and caregivers’ perspectives through narrative medicine
Source: BMC Cardiovasc Disord. 2020 Dec 12;20:522. doi: 10.1186/s12872-020-01809-2 (PMC7733244; doi:10.1186/s12872-020-01809-2)
Supplement: Supplementary file 1 — Additional file 1: Appendix 1 Sociodemographic survey for participants. The Appendix provides the track of thesociodemographic surveys employed to collect data of, respectively, patients, caregivers, andhealthcare professionals involved in the research. [file 12872_2020_1809_MOESM1_ESM.docx]

**Appendix 1**

**1.1. Sociodemographic survey addressed to patients**

1. Age: __________

2. Gender (please select):

- Female
- Male

3. Nationality

4. Residency Region: __________

5. The inhabited centre where you live includes (please select):

- Less than 25,000 inhabitants
- Between 25,000 and 50,000 inhabitants
- Between 50,000 and 250,000 inhabitants
- More than 250,000 inhabitants

6. Marital status (please select):

- Single
- Married
- Separated
- Widowed

7. Do you have children? (Please select)

- Yes
- No

8. You are currently living (please select):

- With one or more familiars
- Alone
- Alone, but I have familiars living next to me
- With a paid assistant

9. Educational level (please select):

- Elementary school
- Intermediate school
- High school
- Bachelor or Master

10. Type of employment (please select)

- Student
- Employee
- Self-employed
- Not working
- Retired

11. How many years have you been living with heart failure? __________

12. How do you consider your condition? (Please select)

- I feel good and have no limitations in physical activity
- I feel good at rest, but ordinary physical activity causes the appearance of symptoms
- Symptoms also appear for less than normal physical activity, but I feel good at rest
- I am unable to perform any activity and have symptoms even at rest

13. What ejection fraction (EF) value do you currently have? (Please select)

- Over 40%
- Under 40%
- I do not know

14. Have you ever been hospitalised due to heart failure? (Please select)

- Yes
- No

15. How often do you undergo medical examinations for heart failure? (Please select)

- Only in case of emergency
- Less than once a year
- Once a year
- Once every six months
- More than once every six months
- Other – please specify: __________

16. On average, how many kilometres do you travel to the treatment centre? __________

17. How many different medicines do you take per day? __________

18. How many individual doses of medicine do you take per day? __________

19. Have you visited other specialist centres other than the current one? (Please select)

- Yes
- No

20. What metaphor would you use to define heart failure? __________

**1.2. Sociodemographic survey addressed to caregivers**

1. Age: __________

2. Gender (please select):

- Female
- Male

3. Nationality

4. Residency Region: __________

5. The inhabited centre where you live includes (please select):

- Less than 25,000 inhabitants
- Between 25,000 and 50,000 inhabitants
- Between 50,000 and 250,000 inhabitants
- More than 250,000 inhabitants

6. Marital status (please select):

- Single
- Married
- Separated
- Widowed

7. Do you have children? (Please select)

- Yes
- No

8. You are currently living (please select):

- With one or more familiars
- Alone
- Alone, but I have familiars living next to me
- With a paid assistant

9. Educational level (please select):

- Elementary school
- Intermediate school
- High school
- Bachelor or Master

10. Type of employment (please select)

- Student
- Employee
- Self-employed
- Not working
- Retired

11. How many years has your loved one been living with heart failure? __________

12. Has your job position changed since you took charge of the person with heart failure? (Please select)

- Yes
- No

13. How many hours a day are you busy assisting your loved one?

- 1-2 hours
- 3-5 hours
- 6-8 hours

14. Which activities do you carry out? (More answers allowed)

- Accompanying him/her to the visits
- Arranging visits for him/her
- Picking up the prescription
- Helping him/her to take medication
- Managing requests for exemption and other bureaucratic requirements
- Accompanying him/her for long trips/travel
- Helping him/her to eat
- Helping him/her get dressed
- Helping him/her to wash himself
- Helping him/her in domestic activities (shopping, cooking, etc.)
- Accompanying him/her on out-of-home errands
- Keeping him/her company
- Taking him/her out for a walk
- Other – please specify: __________

15. How do you consider your loved one’s condition? (Please select)

- He/she feels good and has no limitations in physical activity
- He/she feels good at rest, but ordinary physical activity causes the appearance of symptoms
- Symptoms also appear for less than normal physical activity, but he/she feels good at rest
- He/she is unable to perform any activity and has symptoms even at rest

16. What ejection fraction (EF) value does your loved one currently have? (Please select)

- Over 40%
- Under 40%
- I do not know

17. Has your loved one ever been hospitalised due to heart failure? (Please select)

- Yes
- No

18. How often does your loved one undergo medical examinations for heart failure? (Please select)

- Only in case of emergency
- Less than once a year
- Once a year
- Once every six months
- More than once every six months
- Other – please specify: __________

19. Has your loved one been visited in other specialist centres other than the current one? (Please select)

- Yes
- No

20. What metaphor would you use to define heart failure? __________

**1.3. Sociodemographic survey addressed to healthcare professionals**

**Clinician – General information**

1. Age: __________

2. Gender (please select):

- Female
- Male

3. Specialisation: __________

4. Reference structure (please select):

- Hospital
- University Hospital
- Local Health Authority
- Private practice

5. Region where you work: __________

6. How many years have you been working as a physician? __________

7. Why did you choose your specialisation?

8. With what metaphor would you describe yourself in the exercise of your profession?

9. For you, treating a person with heart failure is: __________

10. What is your perception of the treatment of heart failure onset?

**Patient – General information**

1. Age: __________

2. Gender (please select):

- Female
- Male

3. Marital status (please select):

- Single
- Married
- Separated
- Widowed

4. Does he/she have children? (Please select)

- Yes
- No

5. Does the patient come to visits accompanied by a family member?

- Always
- Often
- Rarely
- Never

6. Educational level (please select):

- Elementary school
- Intermediate school
- High school
- Bachelor or Master

7. Type of employment (please select)

- Student
- Employee
- Self-employed
- Not working
- Retired

8. How many years has the patient been living with heart failure? __________

9. How many years have you been treating this person for heart failure? __________

10. Could you indicate whether the patient has had other heart conditions in the past? If so, could you indicate them? __________

11. The patient has NYHA class heart failure (please select):

- I
- II
- III
- IV

12. The heart failure of this patient is of the following types:

- Ref
- Pef

13. When was the patient's last admission? _____

14. In your opinion, how does this patient perceive – or did he perceive – the severity of heart failure in the early stages of the onset of the disease?

15. With what metaphor would you describe this patient?

16. What metaphor would you use to define heart failure? __________
