# Supplementary material for: The emotional and social burden of heart failure: integrating physicians’, patients’, and caregivers’ perspectives through narrative medicine
Source: BMC Cardiovasc Disord. 2020 Dec 12;20:522. doi: 10.1186/s12872-020-01809-2 (PMC7733244; doi:10.1186/s12872-020-01809-2)
Supplement: Supplementary file 2 — Additional file 2: Appendix 2 Illness plots and parallel charts. Narrative prompts used in the project and specifically designed by the board and the researchers of the ISTUD Foundation. The brackets stand for the space where the participants could write about their experiences.. [file 12872_2020_1809_MOESM2_ESM.docx]

**Appendix 2**

Narrative prompts used in the project and specifically designed by the board and the researchers of the ISTUD Foundation. The brackets stand for the space where the participants could write about their experiences.

|  | **Illness plot for patients** | **Illness plot for caregivers** | **Parallel chart** |
| --- | --- | --- | --- |
| Before the diagnosis/first encounter with the patient | Before the illness […] When I felt […] My heart […] At the beginning I though […] And I decided […] To understand what was happening, I spoke with […] In the meantime I felt […] The people in my life […] At work […] During my spare time […] | Before the illness […] When my […] started to feel bad, I […] At the beginning I thought […] And I decided […] To understand what was happening, she/he spoke with […] In the meantime I felt […] My loved one […] While the other people in my life […] At work […] During my spare time […] | The first encounter with the patient was […] The patient seemed […] While his/her caregiver […] The patient told me […] While his/her caregiver […] So I […] During the communication of the diagnosis I think the patient felt […] While his/her caregiver felt […] So I felt […] I thought […] and I have […] |
| After the diagnosis/first encounter with the patient | When they told me I had HF, I felt […] The first person that gave me the diagnosis was […] And with him/her I felt […] The illness was […] At home […] With others […] I could […] While I couldn’t […] So, I felt […] And I wanted […] | When we discovered that he/she had HF, I felt […] and my loved one […] The first person that gave us the diagnosis was […] And with the physician I felt […] The illness was […] At home […] With others […] My loved one could […] While he/she couldn’t […] So, I felt […] And I wanted […] While my loved one […] | During the next medical examination (s) […] The patient told me […] And his/her caregiver […] The care of HF […] The patient wanted […] In his/her activities […] I thought […] And I have […] |
| Today and future | Today living with HF is […] I feel […] My heart […] Today I can […] The care was […] The relationship with my physician is […] At home […] At work […] During my spare time […] The people in my life […] Thinking about the path before today […] When I think of tomorrow […] I would […] | Today living with HF is […] I can […] My loved one […] I feel […] The care was […] The relationship with the physician of my loved one is […] At home […] At work […] During my spare time […] My loved one […] While the other people of my life […] Thinking about the path before today […] When I think of tomorrow […] I would […] | Today this person […] The patient want […] And today this person can […] The care of HF […] From the relationship with the patient and his/her caregiver I’m learning […] Tomorrow I would like […] Tomorrow I hope […] |
| The experience of writing | Thank you for the time and energy spent. How did you feel writing about your experience? | Thank you for the time and energy spent. How did you feel writing about your experience? | Thank you for the time and energy spent. How did you feel writing about your experience? |
