# Supplementary material for: The emotional and social burden of heart failure: integrating physicians’, patients’, and caregivers’ perspectives through narrative medicine
Source: BMC Cardiovasc Disord. 2020 Dec 12;20:522. doi: 10.1186/s12872-020-01809-2 (PMC7733244; doi:10.1186/s12872-020-01809-2)
Supplement: Supplementary file 3 — Additional file 3: Appendix 3 Sociodemographic data of participants. Sociodemographic data of patients, caregiver, HF specialists who participated to the project and of patients described by physicians in their narratives. [file 12872_2020_1809_MOESM3_ESM.docx]

**Appendix 3**

Sociodemographic data of patients, caregiver, HF specialists who participated to the project and of patients described by physicians in their narratives (grey column)

|  | **Patients (*N* = 82)** | **Patients described by physicians (*N* = 104)** | **Informal Caregivers (*N* = 61)** | **HF Specialists (*N* = 30)** |
| --- | --- | --- | --- | --- |
| **Age** | (*N* = 76) | (*N* = 92) | (*N* = 53) | (*N* = 29) |
| - Mean (range) | 68 (37–98) | 65 (26–90) | 55 (29–79) | 46 (28–65) |
| **Gender** | (*N =*79) | (*N =*102) | (*N =*58) | (*N =*29) |
| - Men | 59 (75) | 77 (75) | 10 (17) | 13 (45) |
| - Women | 20 (25) | 25 (25) | 48 (83) | 16 (55) |
| **Geographical location in Italy** | (*N =*74) | – | (*N =*54) | (*N =*28) |
| - North | 24 (32) | – | 19 (35) | 9 (25) |
| - Center | 20 (27) | – | 13 (24) | 12 (43) |
| - South and Islands | 30 (41) | – | 22 (41) | 9 (32) |
| **Marital Status** | (*N =*75) | (*N =*92) | (*N =*46) | – |
| - Unmarried | 2 (3) | 14 (15) | 8 (17) | – |
| - Married | 56 (75) | 59 (64) | 34 (74) | – |
| - Divorced | 4 (5) | 7 (8) | 3 (7) | – |
| - Widowed | 13 (17) | 12 (13) | 1 (2) | – |
| **Family member** | – | – | (*N =*58) | – |
| - Spouses | – | – | 31 (53) | – |
| - Adult child | – | – | 22 (38) | – |
| - Other | – | – | 5 (9) | – |
| **Educational Level** | (*N =*75) | (*N =*82) | (*N =*54) | – |
| - Bachelor or Master degree | 18 (24) | 17 (21) | 14 (26) | – |
| - High school degree | 19 (25) | 21 (26) | 25 (46) | – |
| - Secondary school degree | 15 (20) | 27 (33) | 7 (13) | – |
| - Elementary school | 23 (31) | 17 (21) | 8 (15) | – |
| - Illiterate | - | 4 (5) |  |  |
| **Work status** | (*N =*75) | (*N =*92) | (*N =*54) | – |
| - Employed | 10 (13) | 10 (12) | 20 (37) | – |
| - Self-employed | 7 (9) | 18 (22) | 6 (11) | – |
| - Pensioners | 53 (71) | 54 (66) | 17 (31) | – |
| - Housewife/unemployed | 5 (7) | 9 (11) | 11 (20) | – |
| - Student | – | 1 (1) | – | – |
| **Medical Specialization** | – | – | – | (*N =*29) |
| - Cardiology | – | – | – | 21 (72) |
| - Internal Medicine | – | – | – | 4 (14) |
| - Geriatrics | – | – | – | 3 (10) |
| - General Practitioner | – | – | – | 1 (3) |

All data are n (%) unless otherwise stated
